# Helpers or Rivals? The Nurse Question

**Published:** 1896-02-22

**Authors:** 


### The Hospital

An Institutional Journal of

The Medical Sciences and Hospital Administration, with

Vol. XIX.—No. 491.] AN EXTRA NURSING SUPPLEMENT. [Feb. 22, 1896.

### Helpers or Rivals? The Nurse Question.

The question whether the trained nurse shall be the doctor's loyal helper or more or less of a rival at the bedside is one which concerns not doctors and nurses merely, but is of first-rate importance also to the patient, and to the progress of medicine. It is quite a new question, as new indeed as the trained nurse; and the unthinking, both among doctors and nurses, have been hitherto very much disposed to believe that in time it will settle itself. But, as a matter of experience, it does not settle itself. On the contrary, the disturbance and discomfort, and even the serious harm brought about by the present state of things, become more obvious and more harassing with every succeeding year.

The *Practitioner* for January, in a very temperate article, gives utterance to the feelings of many of the most sober and judicious members of the medical profession on the subject. "One is continually hearing," says our contemporary, "of nurses scaring old ladies afflicted with 'spasms' with talk about peritonitis and other imaginary horrors; throwing doubt on the diagnosis of typhoid fever made by the family doctor . . . urging resection of the ribs for the treatment of aortic aneurism and tracheotomy for œsophageal cancer, changing prescriptions and treatment," because their own diagnosis differs from the doctor's, and so on.

About the facts, as put forward by our contemporary, there can be no manner of doubt. That the feeling throughout the profession is a very strong one is equally certain. There are two main principles which govern the practical aspects of the case. In the first place, the trained nurse, however long and well she may have been trained, is not medically educated in any sense of that word. In the second, it is plain that in the conduct of medical affairs at the bedside there must be one responsible head, and one only.

In trying to establish a more rational state of things, one has to look about for the causes of the mischief. The first cause is undoubtedly that of sex. The trained male nurse does not give one hundredth part of the trouble that the trained female nurse gives. The woman is egotistical and self-conscious. The "little learning" she possesses is too much for her mental balance. Whilst the man carries the same amount of knowledge easily, and by means of it becomes more clearly aware of his own ignorance, the woman is so puffed up by it that she can see nothing else but what she supposes to be the ignorance of other people. This, however, is not the only cause. There is another, and almost a graver, to which we have called attention before, and that is the attitude of mind which so generally prevails in hospitals on the question of the general practitioner's knowledge and capacity. It cannot be doubted that in very many of the hospitals in which nurses are trained the name of "general practitioner" is one of contempt. Throughout the whole hospital staff, from the senior physician to the most junior house-surgeon, and from the matron downwards among the nurses, the same attitude of mind is more or less universal and more or less pronounced. Of course such an attitude is entirely unreconcileable with reason. Nine-tenths of the resident officers themselves, and almost the whole of the students, are looking forward to general practice as their ultimate goal. Yet even these last, poisoned by the atmosphere of the place, join eagerly in the general contempt. What wonder then if the nurse, being even more susceptible to the influences of environment than her male contemporaries, comes to share unconsciously in the general vicious feeling? In hospital she learns to despise the general practitioner, and when she leaves the hospital to work under that practitioner's control she carries her contempt with her, to the doctor's infinite annoyance and detriment, and to the serious and often fatal injury of his work and of the patient's chances of recovery.

The evil is great, and it is growing. It demands a remedy commensurate with its rankness. The atmosphere of the hospitals in which nurses are trained is what can and must be changed. It is to hospital physicians and surgeons and officials of all kinds that we must look for the remedy. The general practitioner of medicine is the normal "doctor" of the public. The consultant and the specialist are exceptional persons. It is the general practitioner the nurse must be taught to honour; as it is to him she finds by experience she has almost always to look for employment. If hospital physicians and teachers will not defend their brethren in general practice against the ignorant contempt of the foolish nurse, then the general practitioner must do the best he can to dispense with the services of the hospital-trained young woman, and by a process of wholesome starvation bring her to a more reasonable mind.

